# Stakeholder perspectives of family interventions for schizophrenia in Indonesia: a qualitative study

**DOI:** 10.1186/s12888-024-05504-w

**Published:** 2024-01-22

**Authors:** Herni Susanti, Helen Brooks, Budi-anna Keliat, Tim Bradshaw, Dewi Wulandari, Rizky Fadilah, Raphita Diorarta, Suherman, Penny Bee, Karina Lovell, Laoise Renwick

**Affiliations:** 1 Faculty of Nursing, Universitas Indonesia, Kota Depok, Indonesia; 2 Division of Nursing, Midwifery and Social Work, Faculty of Medicine, Biology and Health, University of Manchester, Manchester, UK

**Keywords:** Global mental health, Family interventions, Schizophrenia, Stakeholder views

## Abstract

Mental illnesses comprise the single largest source of health-related economic burden globally and low-and middle-income countries (LMICs) are disproportionately affected. Many individuals with schizophrenia do not receive evidence-based, psychosocial interventions as these are largely unavailable, undeveloped, and under-researched in LMICs. Involving service-users and carers in the design of interventions is crucial to ensure stakeholder needs are adequately addressed by the intervention and to aid successful implementation. We aimed to explore the views and perspectives of different stakeholder groups about the delivery, format, and content of family interventions for people living with schizophrenia in Indonesia as a first step towards developing evidence-based, acceptable family interventions. This study used a qualitative design comprising single stakeholder focus groups. Data were analysed separately using the framework approach incorporating deductive and inductive coding within an existing heuristic framework. 51 participants consented to take part in this study comprising six stakeholder consultation groups including service-users (*n* = 15), caregivers (*n* = 15) and healthcare professionals (*n* = 21). Service users were diagnosed with schizophrenia. Caregivers comprised parents (*n* = 10, 67%), brothers (*n* = 2, 13%), sister (*n* = 1, 7%) and husbands (*n* = 2, 13%). Healthcare professionals were working as nurses (*n* = 6, 29%), doctors (*n* = 5, 23%) or cadre’s (*n* = 10, 48%). Caregiver and service-user respondents had limited knowledge or experience of structured family interventions. There was strong support for such interventions, however, for effective delivery a number of challenges exist in terms of widespread stigmatised views, low expectations for involvement in sharing decisions about care and treatment, views that healthcare professionals are expert and have the authority to delegate tasks to families such as responsibility for ensuring medication adherence and understanding the need to balance the needs of both service-users and families when there are conflicting agendas for treatment. These findings can support the development of evidence-based family interventions for families of those with schizophrenia in Indonesia, as user-informed interventions enhance engagement, satisfaction, and adherence to family interventions.

**Supplementary Information:**

The online version contains supplementary material available at 10.1186/s12888-024-05504-w.

## Introduction

Mental illnesses comprise the single largest source of health-related economic burden globally and low-and middle-income countries are disproportionately affected [1, 2]. Schizophrenia, like many other mental illnesses, often goes untreated in these settings. Indonesia, the world’s 4th most populous nation, has a population reaching almost 280 million people [3]. Most recent estimates place the prevalence of schizophrenia at 1.7 per 1,000 population or approximately 4,539,000 individuals with a treatment gap of almost 40% [4]. The majority of individuals with schizophrenia do not receive evidence-based, psychosocial interventions as these are largely unavailable, undeveloped and under-researched for this population [5].

A confluence of factors underpins the treatment gap but there is a notable absence of human and healthcare resources in LMICs, where the average ratio of mental health professionals to patients is as low as 1:200,000 [6]. Many existing healthcare professionals are not equipped with the skills and knowledge to administer psychosocial interventions. As a consequence, much of the care and treatment needs of people with schizophrenia are transferred to family caregivers, many individuals do not receive even basic care and are solely reliant on family for support [7]. Interest in harnessing the capability of families to utilise their caring role to improve outcomes using evidence-based initiatives is growing [8]. Robust evidence from high-resource settings shows that family interventions, comprising psychotherapeutic and psychoeducational interventions, generate favourable outcomes for individuals with schizophrenia and their families. Evidence shows they are effective for improving individual social functioning, family environment, medication adherence and enhancing therapeutic alliance and importantly, reducing the risk of relapse [9–12]. In low-resource settings, evidence is emerging that family interventions can also deliver positive outcomes for families and individuals with schizophrenia in these settings [13]. However, reviewed evidence suggests that culturally insensitive practices can impede the successful implementation of family interventions highlighting the need to adapt interventions to the culture and context in which they are delivered [14].

Family interventions aim to increase family capacity for dealing with and resolving stressful incidents through encouraging reappraisal of symptoms of illness to enhance family functioning and environment primarily to promote independence for the person who experiences psychosis. The effectiveness of interventions that are based on Western principles delivered in low income settings has been questioned due to differences in cultural expression of emotion [15]. There are some similarities in caregiver burden and emotional strain experienced by Indonesian families of people with schizophrenia and those in Western cultures [16–18] which suggests these interventions may be beneficial in these settings. However, there are differences in appraisals of schizophrenia, cultural beliefs and explanatory models of mental illness across different settings [7, 19, 20]. As such, interventions transferred directly from Western settings without adaptation may not produce equivalent effects in low resource settings [19]. Similarly, there are logistical and demographic differences that may affect the feasibility of delivering such interventions [21].

Illness perceptions and views about the utility and effectiveness of treatments have a role in determining the perceived need and acceptability of interventions for individuals in receipt of treatment but also among those who deliver it [22]. Developing capacity for delivering evidence-based, psychosocial interventions for schizophrenia among non-specialist healthcare workers may be the most efficient way to ensure that interventions are sustainable, ethical and of sufficiently high quality [23]. Evidence shows that when effective interventions are successfully adapted, the acceptability of interventions increases, and people are more likely to engage with help that is offered [19]. As the degree of adaptation is particularly important for improving efficacy [19, 24], there is a need to develop culturally relevant versions utilising robust methods and incorporating the views of wider groups of stakeholders prior to testing [25]. This qualitative study seeks to explore the views and perspectives of different stakeholder groups about the delivery, format, and content of family interventions for people living with schizophrenia in Indonesia.

## The setting

Standard mental health care in Indonesia is limited and there are insufficient financial and labour force resources to deliver evidence-based care. The World Mental Health Atlas 2020 estimates that the proportion of people using mental health services in the past 12 months in the South-east Asian region is approximately 29% [26]. There are variable rates of treatment with national household data providing different estimates depending on the source, ranging from 35 to 85% receiving treatment [27, 28]. Though true estimates of unmet need are currently unknown, many individuals do not receive even basic care and are solely reliant on family for support [7]. There are few reports of interventional studies comprising psychoeducation for families of people with schizophrenia [29–31], but these are not provided routinely in Indonesia. The country has 43 regional mental health hospitals; however, some provinces do not have access to hospitals at all and the psychiatrist-to-population ratio is estimated at 1:200,000, which is far below the World Health Organization’s recommended standard of 1:30,000 [32].

Mental health is becoming a priority in Indonesia with the introduction of a national plan for minimum standards of mental health provision in 2016 [33] defined as one of the 12 healthy family indicators prioritised at a primary care level. The passing of the Health Law into legislation in 2023 [34] also protects the interests of individuals with mental illness and their families, denoting rights to financial, psychological, and social support and to receive treatment without discrimination, placing obligations on central and regional governments to develop and improve mental health services. However, service and treatment provision are patchy, mainly due to low funding, inadequate numbers of sufficiently trained health workers with competing responsibilities and excessively high caseloads among care coordinators for people with serious mental illness. For example, a single nurse in a primary care centre may be in charge of managing 203 health programs e.g. maternal and child nutrition, communicable disease control, family planning, in addition to the mental health program which is the primary source of day-to-day care for people with schizophrenia.

### Aim

The aim of this study is to explore the priorities and preferences for receiving and delivering psychosocial family interventions among key stakeholders in Indonesia including people with schizophrenia, caregivers and family members and healthcare professionals as a first step towards developing evidence-based family interventions for this population [25, 35].

## Method

This study used a qualitative design comprising single stakeholder focus groups. The study was granted ethical approval from the University of Manchester Ethical Review Committee (Ref: 2020-8041-13687) and Universitas Indonesia Faculty of Nursing Ethical Review Panel (Ref: 162/UN2.F12.D1.2.1/PPM2021). Findings are reported in line with COnsolidated criteria for REporting Qualitative research (COREQ) [36].

### Participants

We recruited participants from two areas in Java, Indonesia; Jakarta and Bogor to allow for variation in culture, service infrastructure and organisation, delivery and receipt and urbanisation. We included caregivers or family members if they were aged over 18, living with or spending at least 10 hours per week in face-to-face contact with an individual with schizophrenia who assumed a caring role [37] and were able to provide informed written consent. We included service-users with a diagnosis of schizophrenia or related psychosis and currently receiving treatment in a primary care setting if they were over the age of 18. Healthcare professionals were included if they worked in a primary care setting with responsibility for delivering mental health care. We sampled purposively for gender, age and geographical location to ensure maximum variation within the sampled population [38]. Healthcare workers had specific training in mental health and were responsible for delivering the mental health program within their primary care setting. Having delivered formal, structured family interventions was not a criterion for eligibility for inclusion since services are under-developed in this respect throughout Indonesia and it would be difficult to recruit individuals to get their views on preferences for delivering family interventions. Aligned with evidence that high levels of both code and meaning saturation can be attained with two focus groups, we aimed to conduct two consultation groups per stakeholder group totalling six groups over two sites and checked saturation was reached following data collection and analysis [39]. Participants were recruited using information sessions about the study with primary care workers in the target setting. Recruitment was conducted in primary care settings and invitations were sent directly to potentially eligible candidates. Participant’s capacity to consent and give an account of their views and preferences were evaluated by referring clinicians prior to referral to the study team. Eligible participants contacted the research team following expression of interest, an initial meeting was arranged wherein the researcher’s provided information about participation in the study, provided an opportunity to clarify any uncertainties about participation and obtained informed written consent. Recruitment commenced in October 2021 and was completed in December 2021. Again, having received formal, structured family interventions was not a criterion for eligibility for inclusion in our study. 

### Data collection

**Fig. 1:**
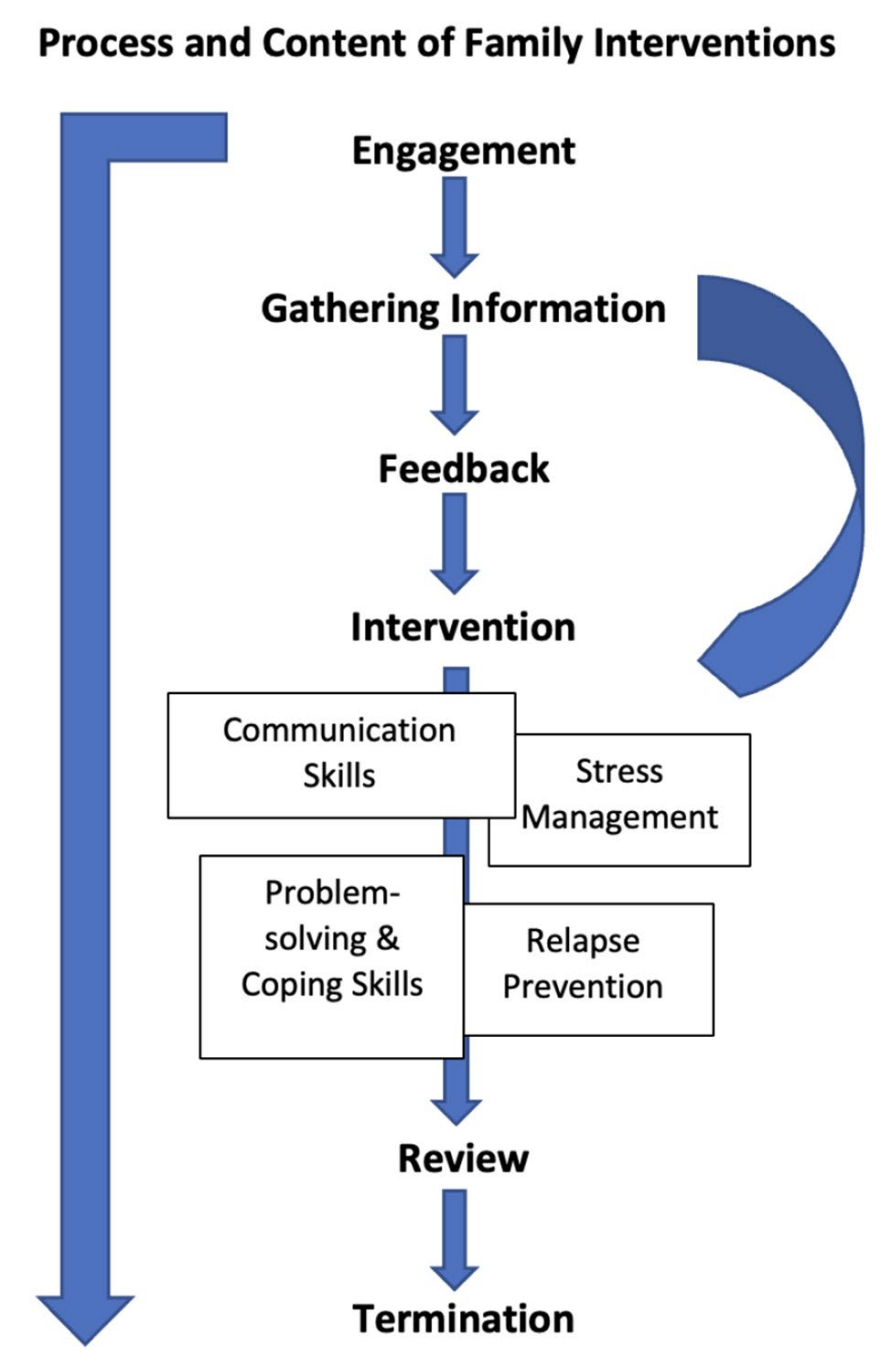
Process and content of family interventions

Participants were invited to attend a focus group that took place online due to pandemic restrictions and groups contained a mix of participants from each location. Focus groups were facilitated by two researchers and a technical assistant and informed by a topic guide to elicit beliefs, attitudes and experiences of mental health service delivery and explore views regarding the components of family interventions and therapeutic outcomes. A semi-structured topic guide (Supplementary File 1) was developed and piloted and adaptations were made to improve the flow of focus group discussions. Demographic information was obtained from participants including age, gender, occupation, education level, marital status, ethnic group and where relevant, number of hours spent caring for individual with psychosis or duration of tenure as healthcare worker. The consultation guide comprised questions and prompts about the following key areas of delivering family interventions: description of family interventions, previous experiences, and views about the need for and importance of family interventions, views about the components of family interventions, perceived outcomes, and preferences for mode of delivery. We used the Barrowclough and Tarrier model of family interventions [40] as the basis for discussions around the components of the intervention. Focus groups also explored individual’s views about factors that helped or hindered delivery of psychotherapeutic interventions to families in this population. The duration of each group was between one and two hours, each group was digitally recorded and transcribed by researchers in the study team into Bahasa. A sample of the transcripts (50%) was anonymised and translated into English and shared with the UK study team for the purposes of supporting the analysis. See Fig. 1 for process and content of family interventions. These data were used to inform the development of culturally adapted, evidence-based family interventions for schizophrenia, the protocol for this study has been published elsewhere [25].

### Data analysis

Data from service-users, family members and healthcare professionals’ groups were analysed separately using the framework approach incorporating deductive and inductive coding [41, 42]. Analysis followed the five stages of framework analysis: familiarisation, identification of a theoretical framework, indexing, charting, and mapping and interpretation [42]. An existing heuristic framework for culturally adapting psychosocial interventions [19, 43] was used and codes were developed within the framework. Codes were also checked across transcripts within the framework to ensure there were no omissions and we considered additional categories for data that did not fit within the framework. Analysis was undertaken manually using Microsoft Excel and coded segments from each transcript were tagged with excerpts linking emerging themes within Excel and denoting the participant type. To ensure consistency of coding LR and HS coded one transcript independently before the study team analysts in Indonesia coded one transcript from each stakeholder group to develop the coding framework. At this point themes emerging from the coding process were discussed among the study team analysts to achieve consensus before proceeding to code the remaining transcripts using the existing framework and gathering raw data segments to illustrate how data were interpreted. Data collection was undertaken by Indonesian researchers and analysis was undertaken by members of both the UK and Indonesian study teams (Authors LR, HS, BAK, TB, DW, RF, RD, S). Data and preliminary analyses were presented to the Research Advisory Group comprising key stakeholders including service-users and healthcare professionals during regular advisory group meetings and views sought to verify data analysis and interpretation.

## Results

51 participants who met the inclusion criteria consented to take part in this study comprising six stakeholder consultation groups including service-users (*n* = 15), caregivers (*n* = 15) and healthcare professionals (*n* = 21). All service-users were formally diagnosed with schizophrenia and had attended both inpatient and outpatient mental health facilities, mainly provided within the public healthcare system. Durations of illness ranged from 2 to 37 years (Mean 17, SD = 11). Caregivers were primarily parents of individuals with schizophrenia; brothers (*n* = 2, 13%), sister (*n* = 1, 7%) and husbands (*n* = 2, 13%). Healthcare professionals were working as nurses (*n* = 6, 29%), doctors (*n* = 5, 23%) or cadre’s (*n* = 10, 48%). See Table 1 for sample characteristics.

**Table 1:** Sample characteristics

		Service-Users	Caregivers	Healthcare Professionals
		n%	n%	n%
Gender	Male	9 (60)	7 (47)	1 (5)
	Female	6 (40)	8 (53)	20 (95)
		Mean (SD)	Mean (SD)	Mean (SD)
Age		41 (8)	60 (10)	41 (10)
		n%	n%	n%
Education	Elementary School	2 (13)	4 (27)	0 (0)
	Junior High School	1 (7)	3 (20)	3 (14)
	Senior High School	10 (67)	7 (46)	5 (24)
	Vocational	0 (0)	0 (0)	6 (29)
	Bachelor Degree	2 (13)	1 (7)	7 (33)

Caregiver and service-user respondents had limited knowledge or experience of structured family interventions, and few described receiving any talking therapies either for themselves or in partnership with their caregivers or families. Drawing on experiences of their involvement with healthcare professionals, service-users and family members believed the purpose of contact was mainly to provide information about symptoms, daily functioning and ensure continued medication adherence. A small number of service-user and caregiver participants had experience of healthcare professionals engaging with them to meet their information and support needs though this was limited, and no participant described receiving any form of intensive, talking therapy. In contrast, healthcare professionals described offering varied psychotherapeutic interventions including psychoeducation, ‘expert’ talks, self-help groups and individual counselling session, mainly underpinned by information-giving and problem-solving techniques with some reference to cognitive approaches. Caregivers, service-users, and healthcare professionals firmly endorsed the need for such an intervention; views about the prospect of receiving family interventions were overwhelmingly positive. Participants perceived several benefits including promoting encouragement, support and hope about meaningful recovery and independence. Healthcare professionals believed structured psychotherapeutic approaches could also alter family perceptions about illness and minimise criticism important for reducing stress and acknowledged that service-user needs should be balanced with the need to improve caregiver wellbeing, mainly to support the function of aiding the individual with schizophrenia.

The results are reported using the headings of an existing heuristic framework which provides an evidence-based, overview of criteria for adaptation of psychological interventions for schizophrenia [19]. Within each framework component, where available, the views, experiences and treatment preferences and priorities are presented alongside constraints, contrasting service-user, caregiver and healthcare professional views and perspectives. See Table 2 for reference to quotations from the transcripts.

**Table 2:** Quotations reflecting participant experiences

Concepts and Illness Models	Stigma and Shame: Self-stigma and Family Stigma “at that time it became more dominant that only the mother continued to talk, if for example she called her child, “My child doesn’t need to be listened to, Mom, he is crazy” like that… so even his own family… his language stigmatizes the same as his family members” N3 “His family paid no special attention, so he was restrained. So the mother restrains her child, the child is not allowed to go out to buy this. So the child is constrained by the mother.” N2 “Most parents feel ashamed because their children are ODGJ so they are locked up like that” N2 “When there’s a family member diagnosed with mental illness or schizophrenia, the family feel worry of becoming the afflicted object of the stigma by the society.” N3 “… the fear of the family’s mental health being affected is very heavy, isn’t it. The first time I cried, ma’am, that time, yes, the first time” K2 Stereotypical Beliefs: Healthcare Professionals “patients who are frequently visited cannot be spoken to… the only ones I talk to are their families, then in my area there are 7 people with ODGJ, and 3 of them have died, only 4 are left, one can be spoken to the patient, the three of them cannot be spoken to…” N4 “The challenges that I imagine is that after the patients recovered……. it is possible that their families will pressure them with lots of new responsibility. In this case, I always remind the families to not force them to do many things at once. Let them progress slowly while continue monitor their medication” N9 Explanatory Models “The health workers in Puskesmas told my child to recite istigfar (seeking forgiveness from God) whenever he gets angry. K9 “We have to remind the patients about our religion so that they remember about God which eventually help them get rid of the hallucination.” K12 Recovery: Support and Inclusion “Yes ma’am, good ma’am for the patient’s own healing process, ma’am. So that he can interact within the midst of society, ma’am” P3 “Family therapy is good, in my opinion, because it can treat families directly and the effect on us is so good. So we can become strong, strong human beings and accepted by society, ma’am” P3 Recovery: Treatment Compliance “The characteristics of Schizophrenia, ma’am, can be cured by taking the right medicine, ma’am” P3 “ I want to give my opinion, ma’am, broadly speaking, this disease can be cured in a fast way, ma’am. As long as you take medication regularly and check with your doctor regularly, ma’am.” P3 “Yes, it is very necessary even though we also often give direction “Mom, actually the child must be corrected, softened” so…” N2 “ the control. but when he is not informed that his recovery is likely he will be negligent, ah lazy to take a lot of medicine like this too.” N7 “taking medicine, now his family because he feels, this family member is already independent, so they forget to control him… forgets to control to check if the medicine has been taken or not…” N3 “the patient at my place in connection with the medicine running out, he will chatter and even throw things, but when we come, then we ask the mother?…Mom why is L getting angry? again…yes the medicine has run out, I haven’t had time to take the medicine…” N7 “ma’am when for example the family can’t listen to us, but usually when something like that happens, for example, the child has a relapse, yes, we remind you, we tell the family this is what, ma’am…what happens if the family also doesn’t care, isn’t obedient, for example….” N3 “I met a family of a patient that said that they cannot force the patient to take medication.” N10 “I think the family plays an important role and have to master the skill of how to communicate with the patients. The families need to talk to the patients calmly and softly. They cannot get angry and stop the patients from talking and sharing their thoughts. And what’s important as well is how the family should remind the patients to take medicine” N9 “We have found some cases of relapsed patients and we observed that it happened because they families are not willing to learn” N14
Family	Family: Role in Recovery “To recover, we not only need to take routine medication but we also we need support from parents and family” P2 “Families need to be informed about our mental health condition. Not only me, I need my mom, dad, and siblings to also understand my illness and condition” P5 “I know one patient who depend so much on his family; his parents and his siblings” N11 Collaboration between Families and Healthcare Professionals “usually I and my husband who visit, well, as… I am told to answer this, ma’am….we are asked about my child’s development until what condition… that means about his health development, his sleeping, whether he takes the medicine, that’s it ma’am. Every time we visit, every time I take medicine, once a month.” K2 “I often tell the doctor…(about disturbing behaviour)…But the doctor was silent, so the doctor just gave me a prescription, ma’am.” K1 “I hope that health workers from Puskesmas can visit my house so I can get to know them better” K7
Communication	Communication Preferences “They always monitor us by whatsapp” K12 “I think both written and direct communication is good. But the important thing is it has to be done by doctors and nurses and done continually.” P2 Shared Decision-making “I think the family plays an important… And what’s important as well is how the family should remind the patients to take medicine” N9
Content	“I wish that I can release my stress and ease my heart after getting this family therapy so that I can continue giving care for my child” K10 “The most important step is the fifth step to make the patients be able to get back together with their family.” K8 “The third step about stress management is the most important one for me. Although the patients may experience relapse, if the carers have a good stress management, I think they can handle the patients and even calm other family members” K7 “Stress management is important because families need to take care of us comprehensively” P3
Cultural Norms and Practices	Religious Practices “For me, family therapy teaches (the patient) about prayers, reciting the holy Quran……”“ K1 “Thank God for me Ma’am… I… my family is not stressed. Alhamdulillah, we always believe in Allah, surrender and while getting treatment I said earlier. And thank God my son has recovered… In sha Allah he will recover.” K2 “We, as family who care for the patient, need to have a stable emotion and I will always remember God and recite istigfar (seeking forgiveness from God) when dealing with him.” K7 “The health workers in Puskesmas told my child to recite istigfar (seeking forgiveness from God) whenever he gets angry.” K9 “We have to remind the patients about our religion so that they remember about God which eventually help them get rid of the hallucination.” K12 “Actually, the religious approach is not only for the patients but also for the family. For example, my wife as the carer, staying close to God will automatically release her stress.” K12 “I expect this family intervention to remind me to pray and recite Quran. And maybe to give me some kind of a special attention.” K1 “In order to care for my brother, what I do is, I need to pray more and remember God even more.” K7 “The second thing to do is, we need to teach the patients about religiosity especially if we are Moslem. This will make them remember about God the Creator. With this kind of therapy, the hallucinations will disappear” K12
Context and Delivery	Format and Mode of Delivery “The obstacle that I usually find is the denial shown by the families. Some families usually deny that their family members have mental illness, so they refuse the help we offer to them. We offered to give them counselling and teach them stress management, but they didn’t want it.” N17 “In our Puskesmas, we have many programs and none of the programs becomes our focus. I am worried that we might not be able to be consistent in giving the family intervention because we also need to run other programs as well. We also lack health workers if compared to the number of patients so we count on our cadres to help us with our tasks. And they even already have so much to do.” N13
Treatment Goals	Family Wellbeing “Family therapy is important for us, family therapy is to change the family’s perspective on things that are negative about mental health. Because families also need information, they need support on how to care for family members who are affected by the disorder. Because when a family member is disturbed, the family doesn’t know what to do. That’s why family therapy is important…” N3 Relapse Prevention “Doing the work of helping my mother at home and outside the house also has to have something to do with it, my relationship with that person, and continuing to take care of my family from what calamity will come to me again, ma’am.” P2 “Yes, I think that therapy is useful so that the patient does not depend on other people. It means that you can be independent here, you can stand on your own… So that the patient knows how this patient lives his life that way, is more useful for society, ma’am, and knows what to do” P6

*Note*: 1 P = Service-user, K = Family member/caregiver, N = Healthcare professional

### Concepts and illness models

#### Stigma and shame: self-stigma and family stigma

Healthcare professionals accounts of families response to having an individual with serious mental illness in the family focused on themes of stigmatisation and feelings of shame. They reported frequently coming up against negative attitudes from family members about treatment and healthcare professionals and reported that treatment refusal was commonplace. While there were several factors noted as specific barriers to improving access to treatments for families, healthcare professionals viewed stigma as a strong and persistent threat to effective treatment delivery as they believed families were less likely to look for and accept available help due to the nature of experienced stigma. They also noted that rejection, discrimination, and isolation was experienced by whole families in response to individuals experience of mental illness. Participants from all stakeholder groups, including families and service-users, recognised the negative impact of stigma but views about the impact of this were less prominent among the latter.

Although families and individuals with psychosis experienced stigma and discrimination in their daily lives as a consequence of the illness and behaviours displayed by service-users, there were also reports that families negative appraisals and treatment of individuals with psychosis stemmed from beliefs about the nature and controllability of the behaviours. These beliefs were reported mainly by healthcare professionals explaining how within families, individuals with mental illness were rejected and excluded by their family members as a result of community responses and reactions to the ill individual and expectations that the family were responsible for managing the illness and behaviours without having the personal resources to do so. Healthcare workers reported encountering families who denied their loved one had a mental illness (*possibly due to different explanations of the behaviour, see below Explanatory Models*) and rejected them, ignored, discredited, or belittled them in their interactions with professionals. Less frequently, they encountered ‘pasung’ where families secluded or restrained the individual with schizophrenia, restricting their movements through community confinement because of community pressure to manage and control bizarre and inappropriate behaviour.

#### Stereotypical beliefs: healthcare professionals

Healthcare workers narratives encompassed values of kindness and compassion towards people with psychosis but they also voiced frustrations regarding the lack of support service-users received from others, mainly their families, to maintain treatment. Professionals displayed stereotypical beliefs and negative views about service-users and the potential benefit that treatment could provide. Terminology used, such as laziness and disobedience, indicated underlying beliefs about the controllability of the symptoms experienced by service-users. There were also instances where healthcare professionals described withdrawing their support from service-users and families when they considered their symptoms untreatable. Some cases where individual service-users and families displayed high care and treatment needs healthcare professionals justified disengagement based on these needs and the limitations of their own practice representing underlying hopelessness about illness trajectory among healthcare professionals. Paternalistic attitudes among professionals were evidenced in reservations expressed that through providing family interventions and supporting individual recovery, families would pressure service-users to work and contribute financially which may exacerbate symptoms and jeopardise recovery. Negative attitudes from healthcare professionals towards family roles and involvement were also evidenced in some workers narratives.

#### Explanatory models

Explanatory models among family members and service-users were dominated by spiritual beliefs and attributions of religious beliefs to mental illness. Families, and service-users to a lesser degree, believed that spiritual forces explained the process of recovering from symptoms such as hallucinations, believing that praying and engaging in religious practices was a preferred approach to rid family members of unwanted experiences. They reported these types of approaches were also promoted by healthcare professionals during their interactions. Service-users also reported other commonly held beliefs about illness causes e.g., contagion/infectious disease, supernatural forces important for informing the content of family intervention psycho-education components.

#### Recovery: support and inclusion

Service-users and families coalesced on concepts of healing and recovery, prioritising this process as a personal experience, and embodied in language about recovery and healing beyond the absence of symptoms. Varied concepts of the meaning of healing and recovery were expressed in service-user narratives stemming from both personal and clinical recovery narratives including symptom reduction, regaining enthusiasm for life, staying active both on a personal level and within family life, gaining employment and returning to independence. Some service-users believed recovery was signalled by returning to a functional state equivalent to premorbid levels or what they considered normal attainment, and this was particularly prominent for families also. Engendering hope for the future was an important aspect of the role of healthcare professionals and this was echoed in service-user narratives about the function of family therapy as a means of driving enthusiasm and support from families about future recovery that enabled service-users to heal *(see Treatment Goals)*.

#### Recovery: treatment compliance

Service-users, to some degree, conflated notions of healing with being cured presuming they continued to take prescribed medicines and engage with appointments with doctors. In general, adherence to medicines and maintaining good therapeutic relationships with healthcare professionals were valued as mechanisms for obtaining recovery but were underscored by recovery narratives around making amends for past behaviours and having been ill from service-user perspectives and the need to be fixed from healthcare professional perspectives. Strong narratives emerged from both families and healthcare professionals of the need to compel individuals with schizophrenia to take prescribed medication denoting prevailing paternalistic approaches to mental health care delivery.

### Family

#### Family: role in recovery

Service-users consistently endorsed views that their families were central to their recovery, providing support for taking medication and encouragement and reassurance in gaining independence. Not only parents but spouses, siblings and extended family members had considerable involvement in supporting individuals with psychosis. The positive role of family was a consistently strong theme among service-user narratives and many felt positive about the involvement of family in their care, not only to aid the person with psychosis but as a source of support for family members who may experience additional burden due to their responsibility. Service-users, however, expressed apprehension that families would view interventions as burdensome and attending or scheduling sessions may not be prioritised over other caring roles. Family members supported this, noting a high burden of care generally, not only for the individual with mental illness but other family members with care needs such as ill spouses, children, and grandparents that could compromise their ability to prioritise the needs of the family member with schizophrenia.

Healthcare professionals also emphasised the essential role of family members in recovery but reported negative experiences attempting to support families in caring for the individual with psychosis. Professionals reported that families reluctantly engaged with formal help available and had difficulty responding to the needs of the person with psychosis due to denial of the illness and alternative explanations of illness. Professionals were sceptical that the needs of the individual with psychosis would be prioritised over the needs of the family as a whole and competing demands for time and resources, including financial resources, the need to maintain an income and the rising cost of treatment and medicines for ill individuals.

#### Collaboration between families and healthcare professionals

Families expressed gratitude with initial support received from healthcare services in terms of information about the diagnosis and support during initial crisis and prioritised this as a component of family interventions. They also expressed frustrations regarding a lack of support from healthcare professionals, reporting feeling uninformed and excluded from decision-making about treatment and reported an expectation that families are responsible for monitoring medication and ensuring engagement with mental health services. Indeed, narratives emerged of a shared understanding among families and healthcare professionals that medication monitoring is a key role for family members and sometimes appointments for outpatient review are made directly with the family without the individual with schizophrenia. Families described how they had accepted responsibility for the individual with psychosis and this was operationalised as managing care on a day-to-day basis. Despite this, family members believed they lacked the resources, skills and support to manage symptoms and illness behaviours and were frequently unprepared for the role they had assumed. They believed they needed the ‘authority’ of healthcare professionals to compel individuals with schizophrenia to comply with instruction and in this sense, felt unsupported by healthcare professionals when the person with psychosis did not follow the instruction of family or professionals. On the other hand, evidence from professional narratives suggested they viewed families as uncooperative, resistant to instruction and disobedient displayed underlying negative attitudes towards families and views that the professional’s role formed a didactic problem-solving stance rather than collaborative and equal partnership.

### Communication

#### Communication preferences

Service-users and families appreciated being given information about psychosis and recommended treatments, reporting many positive experiences with healthcare professionals in this respect. Both groups reported feelings of being valued and learning practical ways of problem-solving citing the value of knowledgeable professionals. Culturally sensitive ways of approaching family interventions were also important for service-users, prioritising the importance of professional values such as displaying kindness. Different respondents expressed different preferences for communication as a component of family interventions in different formats and modalities. Families more often expressed a preference for written materials to accompany information given during sessions. Service-users reported they currently communicated with healthcare professionals via digital applications i.e. WhatsApp and appreciated that their progress was monitored in this way.

#### Shared decision-making

There were also cultural ways of communicating between healthcare professionals, families, and service-users more consistent with collectivist principles; it is customary for people with mental illness to be accompanied on healthcare appointments by family members and for consultations to be conducted including all parties. In terms of planning care, both service-users and families tended towards adopting a passive decision-making role, delegating responsibility for decision-making to healthcare professionals. Professionals were viewed by both groups as knowledgeable experts with low expectations that individual personal preferences and needs will be taken into account. Service-user narratives indicated they sometimes delegated responsibility for communicating with healthcare professionals to family members and this is culturally acceptable practice. Healthcare professionals in turn expressed expectations that families should maintain oversight of the individual’s condition and treatment and assigned responsibility to families for supervising and regulating their behaviour.

### Content

Families, service-users, and healthcare professionals were in broad agreement regarding the ingredients of family interventions based on an existing cognitive-behavioural model of family interventions including problem-solving, psychoeducation, communication skills, stress management and coping skills enhancement. Service-users emphasised the importance of the initial assessment, including families understanding of the illness, their burdens in taking care of family members with mental disorders, how families find solutions for their own problems, how mental illness affect the patients and the families and about the strengths of the family. Families understanding of the term needs within the context of the assessment of relatives and service-user’s needs differed culturally and contextually from those articulated in cognitive behavioural models of family interventions. Needs in this context refers to material resources such as finance, housing, employment due to cultural meanings and successfully restructuring problems identified as needs will require strategies to provide a relevant outline of areas that may be targeted therapeutically during the course of the intervention. Service-users were also largely in agreement that they should attend some sessions with their families. Family members prioritised different components of family interventions based on their own experiences although stress management was highlighted among multiple narratives from both families and service-users as a specific priority. Families also expressed a strong desire to gain new techniques and skills and were concerned that existing treatments were focused on ensuring service-users were compliant with prescribed medicines and this was reinforced in interactions with healthcare professionals.

### Cultural norms and practices

#### Religious practices

Observing religious practices and attending to spiritual needs were strong themes among family and patient narratives, particularly as a method of countering stress in both service-users and family members. Encouraging religious practices is viewed as an important aspect of supportive, psychological interventions and how belief in a higher power acted as a buffer towards the negative psychological effects of stressful experiences. Some narratives demonstrated how some families religious backgrounds shape health beliefs and attributions to illness underlining the availability of alternative and wider sources of care and support from non-traditional sources. Families also reported how these practices were embedded in existing care encounters. Family accounts especially reflected the importance of faith and religion as central features of Indonesian society and culture and dominated the discourse on culture-specific practices and norms from caregiver perspectives.

### Context and delivery

#### Format and mode of delivery

All groups of participants believed adaptations to settings were necessary to ensure feasible delivery of family interventions. Service-users and families expressed concern that family interventions would increase caregiving burden and add strain to existing roles. Service-users were particularly concerned about increasing carer workload and family members were concerned that they would be unable to attend all sessions due to competing responsibilities. Family members were in broad agreement with the mode and duration of delivery, agreeing upon ten sessions to be conducted with individual families. They suggested workarounds that would facilitate individual attendance based on existing schedules and priorities such as conducting the intervention at home or having shorter sessions. In contrast, service-users were undecided about whether the interventions should take place at home or in the primary care centre.

Healthcare workers also presented diverse opinions but, like family members were concerned about stigma and involuntary disclosure if interventions were provided in the family home. Despite this, most families wished to have family interventions in their own homes mainly to preserve privacy but also for pragmatic reasons. Healthcare professionals posited advantages and disadvantages of conducting interventions in patients own homes; in favour of the latter, family interventions could conceivably be delivered alongside existing programmes that were already delivered in the community and may reduce the likelihood of further stigmatising families difficult to engage and fearful of medical environments. However, solutions were raised in the context of professional’s concerns about the feasibility of delivering family interventions that were intensive in nature consistently, i.e., individual families and multiple sessions with existing workloads, competing demands, staffing resource shortages and a lack of skilled and trained staff to deliver existing programmes.

### Treatment goals

#### Family wellbeing

Families regarded psychosocial interventions of any type highly and valued the advice and support of healthcare professionals particularly near to the onset of illness when their information needs were at their highest. Professional input thereafter was minimal and mainly entailed gathering psychiatric history and evaluating progress. Families were clear that their experience so far with healthcare workers did not focus on reducing their stress or targeting their own wellbeing despite expressed needs for psychosocial support. Families emphatically expressed their aspirations that family interventions would encompass a wider range of treatment goals to promote family wellbeing. Families sought greater harmony in their relations with their loved ones as a priority but also expressed a desire to collaborate more closely with healthcare professionals. Service-users also envisaged a more extensive range of positive benefits that could be achieved through engaging families in psychosocial interventions primarily via enhancing recovery. Service-users strongly emphasised the need for occupation, both therapeutic and gainful employment which they believed afforded opportunities for greater social inclusion and recognised the latter was often provided in a watchful and supportive way by families within the home.

#### Relapse prevention

Relapse prevention was a prioritised intervention and fundamental component of family interventions from the perspective of service-users, families, and healthcare professionals. Service-users believed families could monitor individuals to detect relapse earlier and facilitate earlier support and help to prevent relapse important for building relapse prevention strategies as an intervention. Professional and families’ views focused on prevailing obligations to ensure continued medication administration to prevent relapse. Service-users expressed a desire to work, live and engage in community life without constraint but with a greater focus on becoming a useful member of their family and societal units. From professional’s viewpoints, the treatment goals of family interventions were to alter negative cognitions and improve knowledge, improve self-confidence, adherence to medicines, enhance social support, greater family wellbeing and negotiation of agreement between family and service-user about the nature of the problem and treatment goals going forward.

## Discussion

In this qualitative research study exploring the priorities and preferences of service-users, caregivers, and healthcare professionals for family interventions for people with schizophrenia we found participants were overwhelmingly positive about the prospect of receiving family interventions. Drawing on an existing heuristic framework, our analysis elucidates the key constituents and culturally relevant therapeutic targets of family intervention to optimise the delivery of these interventions in this setting. Presently, these settings are limited by scant access to credible evidence regarding effective, evidence-based psychosocial interventions for serious mental illness [23] and there is a pressing need to reduce the treatment gap by expanding mental health service provision making effective treatments available for those most in need [44]. Our findings show that families have minimal experience receiving family interventions and many were unaware of the availability of these types of psychosocial interventions which is consistent with intelligence that these types of interventions are not routinely provided throughout Indonesia. Indeed, the vast majority of people with schizophrenia residing in low-resource settings receive no evidence-based psychosocial treatments [45].

Caregivers place high value on receiving support through psychosocial interventions in low resource settings as shown in this study [21]. Despite this, evidence from high resource settings shows that there is poor engagement with existing interventions, high attrition rates, and patchy delivery across different settings [46, 47]. Some authors suggest that differences between collectivist and individualistic cultures, where family involvement in care and treatment is greater in the latter may translate into higher levels of engagement, retention and acceptability of family interventions in low resource settings [48]. However, there must be consideration that enthusiasm for family interventions among our family participants may be an artefact of our sampling strategy as self-selecting individuals may have a greater inclination and optimism towards family involvement in care. This underlines the need to further evaluate the acceptability and feasibility of family interventions in these settings to explore actual recruitment and retention rates in these settings exploring perceived barriers and facilitators to implementation.

An additional factor that may influence engagement and retention in interventions is the degree to which the recipients view the interventions as useful, acceptable, and effective for meeting their needs. There is evidence that families contribute positively towards the wellbeing of people with schizophrenia, particularly if they themselves are actively supported by psychoeducation [49–51]. However, there is an extensive evidence base for the effectiveness of family interventions which have historically been more oriented towards improving service-user outcomes. An alternative approach, and one which we have adopted here, is to enhance the fit of evidence-based treatments to those who will use them given that delivering acceptable interventions is necessary but not sufficient to deliver effective interventions [22]. For multiple stakeholders, the relevance of the content, the context for delivery and the quality of the intervention and family-therapist interactions all have implications for the acceptability of and satisfaction with the intervention. This is important, as evidence suggests that service-users are more likely to continue in treatment and benefit from improved outcomes if they deem the intervention delivered as an acceptable one [52]. This principle may extend to family members as these types of interventions can also be effective for reducing family distress [12] reducing mental health morbidity, perceived burden and negative experiences of being a carer [53].

Our study did reveal potential barriers to participation among caregiver and service-users perspectives, in that families experience multiple conflicting stressors, like other illnesses of family members and lack of family understanding and support for mental illnesses, such that prioritising the treatment of a person with schizophrenia within the family context is likely to be challenging. These barriers are consistent with reviewed evidence among families of people with early psychosis in mainly high resource settings [54]. Problems with uptake, however, are compounded by issues of poverty, low rates of education and language literacy and lack of available caregivers [21] in low resource settings. Carer reluctance to attend family interventions may also be linked to perceived discrimination and the impact of stigma was most pronounced as a barrier to effective care in our study.

The fear of stigma and discrimination formed a prominent disincentive to participation in family interventions which is consistent with reviewed evidence that stigma is associated with reduced help- and treatment-seeking behaviours [55]. Evidence that stigma impacts life circumstances including opportunities for education, employment, housing, social and romantic relations [55–57] is congruous with our findings that individuals with serious mental illnesses are excluded from social opportunities and ostracised from their communities. These issues were evident in our findings, notably that the burden of schizophrenia became a source of stigmatisation for the whole family and negatively affected each individual family member. Illness duration and the extent of recovery achieved impacted on families experience of community acceptance, with families whose loved ones had extended periods of recovery and did not exhibit bizarre or unusual behaviour affected less by exclusion from society. Conversely, families whose loved one was acutely unwell or displayed aggressive or violent behaviours, experienced societal pressure to conceal or control the behaviour of individuals to avoid being publicly shamed [58]. Extending synthesised qualitative evidence of families views of stigma [58], our findings suggest that there are cultural factors at play as few service-user and caregiver participants described overt stigmatisation yet consumer views and attitudes were strongly linked with deeply entrenched cultural ways of understanding mental illness. Professional accounts of active discrimination within families is consistent with reports of pasung driven by myths and misconceptions among the general population and the families response to community pressure to isolate individuals from society [59].

Importantly, poorer access to and engagement with professional and therapeutic services is linked with stigmatised views and attitudes [55]. There was a key concern that families attendance at psychosocial interventions would further stigmatise them within their communities [21]. Considering this, modifications to content or delivery format are key to mitigate against further treatment avoidance and optimise the acceptability of family interventions. Service-users and families, felt that delivering the interventions individually in their homes would protect them from involuntary disclosure and subsequent stigmatisation. In direct contrast, healthcare professionals voiced concerns regarding the feasibility of implementing intensive interventions with current levels of resourcing suggesting a creative but comprehensive and considered approach to delivering these interventions in the future will be required including evaluation and development of effective wrap-around implementation strategies and methods to increase demand and uptake of effective psychosocial interventions.

A key finding emphasised growing awareness among families and service-users that antipsychotic medication is inadequate to address the complex social, health and economic burden of having a mental illness and the consequences for families. Despite this, service-users and families prioritised medication and medicines management as the mainstay of interventions for enhancing and ensuring recovery. These narratives were linked with underlying constructs around social control and adhering to social norms and expectations, particularly for service-users. Families were often surrogate decision-makers and service-users delegated responsibility for decision-making to family members which may reflect high levels of interdependence observed in Asian families. Service-users are not viewed as autonomous decision-makers and paternalistic attitudes of healthcare professionals’ consistent with medical models of care views patients as passive subjects who are unable to make their own decisions. This view extends to family involvement in decision-making opposing the principles of recovery.

Healthcare professionals delegated responsibility for ensuring adherence to medicines to family members and expected them to execute and act on the direction of the professionals who were considered experts. To some degree, growing frustration and discontent among family members was evident as they believed they lacked the personal resources and authority to execute these plans amid managing other roles and responsibilities and overcoming their own psychological distress. This served to accentuate the challenges that can arise in meeting the needs of both service-users and families when there are conflicting agendas for treatment [60] recognising that meeting the needs of both groups concurrently may not be achievable [61]. Interestingly, service-users were predominantly supportive of family involvement in their care and their perspectives did not reflect commonly reported challenges to delivering family-focused care in Western nations such as difficulties around safeguarding confidential patient information and protecting individuals right to alter consent provision in decision-making throughout treatment stages [62]. Nonetheless, reconciling consumer preferences may be important for engagement with and adherence to these treatments to enhance consumer satisfaction and improve outcomes [63]. Extending this idea, involving service-users and carers in the design of interventions is crucial to ensure stakeholder needs are adequately addressed by the intervention and deliver successful implementation [64]. These data were used to inform the development of culturally adapted, evidence-based family interventions for schizophrenia and develop training materials for family interventions in primary care settings, the protocol for this study can be found here.

### Strengths and weaknesses

These findings are strengthened by implementing robust data collection procedures and the use of an existing heuristic framework for analysis of data obtained. Data were collected by Indonesian researchers with explicit and detailed protocols devised and supervised by an experienced UK/Indonesian research team. In addition, data collectors were trained in implementing focus group techniques by experienced researchers during in person and online sessions. In this study, we implemented online focus groups to mitigate against local pandemic restrictions. Online focus groups can be limited by the lack of information of the environment, non-verbal behaviours, difficulties implementing risk procedures and technical considerations [65, 66]. However, we found this measure particularly important for ensuring recruitment of an appropriate sample and recruiting vulnerable individuals from remote, marginalised areas [67] who would have been otherwise excluded which enriched our sample. We did experience technical difficulties at times, but this was mitigated against using additional facilitators with specific roles in assisting those with the use of this technology. We acknowledge that gathering data from different sources or utilising different methods can strengthen the credibility of findings but decided not to do so based on the research question posed, the exploratory nature of our study in this context and setting and taking into account that distinct research methods and sources may not produce comparable findings. The findings of this research have been evaluated in the context of limited available evidence to support the delivery of psychosocial interventions in low resource settings. Three systematic reviews including one meta-analysis report on the feasibility and effectiveness of varied types of interventions, all drawing similar conclusions regarding the present scarcity of evidence to support the development and delivery of interventions to target the complex social, economic and health needs of those affected by serious mental illness [21, 68, 69]. As such, this qualitative work extends our limited understanding of consumer perspectives of psychosocial interventions to support evidence-based intervention development. Further research regarding optimal implementation from wider stakeholder groups may be beneficial to support the design of the proposed family intervention alongside implementation strategies. Lastly, the feasibility and acceptability of these interventions tested in naturalistic conditions will further assist in determining the utility of such interventions in low-resource settings.

## Conclusion

Our study indicates that family interventions for schizophrenia is strongly supported among caregivers and service-users in Indonesia despite challenges to effective delivery. Further exploration of implementation, feasibility and acceptability in situ is required to develop effective, culturally relevant evidence-based family interventions in Indonesia.

### Relevance for clinical practice

The findings of this research suggests that family interventions may be an acceptable intervention for people with schizophrenia and their families in Indonesia. However, there are a number of factors that need to be considered in the development to optimise cultural relevance and successful implementation including stigmatisation of individuals and families, lack of resources for implementing intensive interventions and prevailing paternalistic practices among healthcare professionals.

### Electronic supplementary material

Below is the link to the electronic supplementary material.

Supplementary Material 1

## Data Availability

Complete transcripts cannot be made available due to the potentially identifying nature of these data and public access is restricted. Relevant data are available upon request subject to compliance with ethical permissions at the host institutions. Interested individuals may contact Herni Susanti (herni.susanti2017@gmail.com) or Laoise Renwick (laoise.renwick@manchester.ac.uk) to request data.

## Declarations

### Reflexivity statement

Focus group discussions were conducted by Indonesian researchers from among the study team. Data collectors (HS, BAK, S, and DW) and analysts (HS, BAK, S, DW, LR and HB) comprise experienced nurse academics and doctoral students. Several researchers among the study team have clinical backgrounds but none were known to the participants and measures were taken during interview to ensure researchers professional backgrounds did not influence participants willingness to share experiences.
