# Supplementary material for: Stakeholder perspectives of family interventions for schizophrenia in Indonesia: a qualitative study
Source: BMC Psychiatry. 2024 Jan 22;24:59. doi: 10.1186/s12888-024-05504-w (PMC10804701; doi:10.1186/s12888-024-05504-w)
Supplement: Supplementary file 1 — Supplementary Material 1 [file 12888_2024_5504_MOESM1_ESM.docx]

**Appendix 1: Stakeholder Group Consultation Guide**

1. **Introduction and Description of Family Interventions**

Thank you for taking the time to talk with us today. (Outline the distress and risk protocol and reiterate audio-recording, purpose and how the information will be used and stored. Outline housekeeping /how to interrupt on Zoom, breaktimes etc.)

We are interested in hearing your views about talking therapies for families of people with schizophrenia. Family interventions are talking therapies that involve providing education, counselling, and coping skills for families who care for someone with psychosis. The intervention starts with a session where the family speaks to the therapist about their situation and their needs. This is followed by sessions on stress management, goal setting and is delivered using methods that involve active participation e.g. role play, guided practice and self-monitoring. The other key components of the intervention include problem-solving, communication therapy and education about the illness, treatments, and outcomes.

1. **Perception of Need and Importance**
   1. What do you think about family therapies?
   2. How is family therapy different from the care you currently receive?
   3. What aspects of family therapy would be most helpful for people in your situation in Indonesia? What do you think would help you most?
   4. Which aspects would least help you in your situation?
   5. What would get in the way of you attending family interventions?

- Structural issues such as transport availability or cost, distance to travel, accessibility or availability of the service required, lack of resources within hospital, waiting times for services, language barrier, lack of interpretation services.
- Education: not knowing what entitled to, not knowing where to go or how to get there, not understanding how medical care works or which service to access.
- Economic: cost of travel, cost of healthcare, cost of medicine, lack of health insurance, prioritisation.
- Cognitive appraisal: fear of negative evaluation by others, fear that intervention is not confidential, perception help is not needed
  1. What would make it easier for you to take part in these interventions?
  2. What change would you like to see in mental health services that would have the biggest impact on your situation?
  3. Are there any barriers to the use of these types of interventions in Indonesia?
  4. Are there any things that make it easier for people to the use of these types of interventions in Indonesia?

1. **Outcomes and Delivery**

If these interventions were delivered in your area, how would they be best delivered?

- 1. If you had attended this type of therapy, what kind of benefits would you expect to see? E.g. improved coping, understanding the illness better, having a better relationship with your loved one
  2. What characteristics do you value in the health professionals or what helps/hinders rapport with a healthcare professional?
  3. What do you think about filling out outcome measures to help us see the effects of the treatment? (Use KOP, PHQ-9 and GAD as examples) How would we do this best, paper forms or using an online survey?
  4. This type of treatments is usually delivered in 10 sessions between 3 and 12 months? What are your thoughts on this?
  5. Each session is usually 1-2 hours? Do you have any comments on this?
  6. Who should be involved in therapy, service-users, family members? what about anyone else?
  7. The therapy could be delivered in different locations such as the primary care centre, your home, or a community centre. Where do you think it would work best and why?
  8. Is there any particular issues with language, language we should or shouldn’t use? Think of the word’s schizophrenia and psychosis – is it appropriate to use these terms? What about the term carer and service-user?
  9. What can we do to make sure people attend the intervention until the end? Is there anything that might put people off?
  10. What kind of information do we need to provide before and in-between sessions to make sure people are informed?
